# Nutriepigenetics in Skin Homeostasis: Molecular Mechanisms of Honey-Mediated Chromatin Remodeling in Non-Healing Ulcers

**DOI:** 10.3390/biom16091272

**Published:** 2026-09-03

**Authors:** Elia Ranzato, Simona Martinotti

**Affiliations:** DiSIT—Dipartimento di Scienze e Innovazione Tecnologica, University of Piemonte Orientale, Viale Teresa Michel 11, 15121 Alessandria, Italy; simona.martinotti@uniupo.it

**Keywords:** chromatin remodeling, epigenetic memory, epithelial–mesenchymal transition, nutriepigenetics, wound healing

## Abstract

Traditional wound therapies continue to be predominantly exogenous and address extracellular causes of the pathology without addressing the impaired function of cellular pathways that are trapped in the state of constant inflammation. The present review explores a novel putative nutriepigenetic framework, discussing how the honey matrix could act as a proposed modulator of the altered epigenetic landscape in non-healing ulcers. Honey contains a complex mixture of bioactive agents (polyphenols, flavonoids, and plant-derived xenomiRs) that are hypothesized to interact with multiple chromatin control points simultaneously. We discuss models wherein honey-induced aquaporin-mediated H_2_O_2_ influx and intracellular calcium transients may correlate with SIRT1/SIRT6 modulation and CRM1-mediated nuclear export of Class IIa HDACs. This review evaluates whether such multi-target signaling could foster chromatin relaxation to support gene expression required for cell migration and tissue remodeling.

## 1. Introduction

Non-healing, chronic wounds such as diabetic foot ulcers, venous stasis ulcerations, and pressure sores pose a growing challenge to health care systems worldwide [1]. In developed countries, the prevalence of these conditions is about 1% to 2%. The treatment of such wounds, however, can require 3% of all healthcare expenditures. The reason for this problem is a combination of systemic disorders, including ischemia of affected tissues, presence of bacteria, oxidation, and metabolic disorder due to diabetes or aging [2].

The traditional measures for addressing this issue include the use of innovative interactive dressings (hydrocolloids, alginate dressings, matrix dressings with silver ions), negative pressure wound therapy, or growth factor therapy [3]. However, the conventional approach cannot address the problem effectively. As a result, the wounds remain open, allowing infections, necrosis, and, eventually, amputations of limbs.

The primary cause of this treatment failure is due to the inherent reactive nature of conventional therapies which target extracellular influences such as hydration, bioburden, or necrosis but ignore completely the intracellular machinery of the dysfunctional cells. In the case of chronic ulcers, peripheral keratinocytes and dermal fibroblasts are alive but functionally stunted [4]. Immobilized in a constant hyperinflammatory condition, they fail to respond to any physiological stimuli which would normally drive tissue regeneration and repair processes. This cellular arrest is driven by a complex, multifactorial microenvironment involving persistent bacterial biofilms, excessive proteolysis, localized hypoxia, and advanced glycation end-products (AGEs). Within this multifactorial network, altered epigenetic mechanisms represent one interactive layer rather than an isolated single cause.

Hence, this inability of conventional medicine to treat such wounds necessitates looking into naturally complex bioactive agents [5,6]. Honey serves as an excellent example of such a biochemical matrix which simultaneously targets several cellular pathways. Historically, honey was physically and chemically effective in wound healing because of its high osmolarity and thus draws fluids to initiate autolytic debridement, while its low pH level ensures an environment unsuitable for microorganisms [7]. However, it should also be noted that the sustained production of low levels of hydrogen peroxide via glucose oxidase and phytochemicals provides a wide-spectrum antibiotic defense.

Nevertheless, modern studies have found evidence that the use of honey has even greater beneficial effects on the healing of a wound site [7,8,9,10]. Various bioactive fractions of the matrix can penetrate through the cell membranes and engage with their nuclear functions. Thus, there emerges a new approach for wound treatment: the use of honey as a nutriepigenetic factor.

Rather than being used as only a passive protective layer, honey is an active agent whose complex of biologically active components (flavonoids (chrysin, quercetin, apigenin), phenolic acids, stable plant xenomiRs) can alter host DNA methylation, histone tail modifications, and post-transcriptional gene silencing in affected skin cells [11].

Considering the above-mentioned properties of honey, this work reviews the interactions between honey-induced cellular signals (changes in oxidation state and intracellular calcium concentration) and epigenetic alterations, which are reflected in remodeling of nuclear chromatin structure. It discusses how honey breaks up epigenetic silence that occurs in chronic wounds. In conclusion, this review proposes a nutriepigenetic framework to evaluate honey not as a targeted therapeutic editor, but as a complex, putative matrix that may influence local chromatin accessibility.

## 2. Epigenetic Landscapes in Wound Healing and Skin Homeostasis

Before examining the specific biochemical components of honey, it is fundamental to clarify that its potential biological effects do not apply broadly to all honey varieties.

Due to significant botanical and geographic variability, honey composition is highly variable. Therefore, any proposed epigenetic interaction is strictly contingent upon the concentration and bioavailability of specific bioactive constituents present medical-grade honey formulations rather than being a universal feature of honey as a whole.

### 2.1. Epigenetic Control of Healing Phases

Physiological wound healing entails several interrelated phases that include the inflammatory, proliferative, and tissue remodeling phases [12]. Shifts from one phase to another are influenced by the changes in transcription processes of the cells in the skin. This is facilitated using epigenetics which form the basis of regulation in physiological wound healing. The initial inflammatory phase requires prompt transcription of pro-inflammatory cytokines such as TNF-alpha, IL-1beta, and IL-6 by macrophages and neutrophils [13].

This takes place because of the activities of histone modifiers that specifically target certain promoter regions. The activities of histone acetyltransferases (HATs) such as p300/CBP lead to the modification of H3K9ac (acetylation of lysine 9 on histone H3) and H3K14ac (acetylation of lysine 14 on histone H3). Through the activities of histone acetyltransferases, there is a reduction in the charge of histones causing chromatin decompaction. There is also trimethylation of lysine K4 on histone H3 through the action of histone methyltransferase MLL1 [14].

For the wound microenvironment to shift to the proliferative phase, these inflammatory genes need to be silenced in order not to destroy the tissues. This can be achieved through the regulation of these genes by an epigenetic shut down process. In this case, the recruitment of Histone Deacetylases (HDAC), such as HDAC1 and HDAC2, takes place to deactivate these activators, and LSD1 Histone Demethylase, which gets rid of tri-methylation of lysine 4 on histone (H3H3K4me3) [15].

In addition, the Polycomb Repressive Complex 2 (PRC2), which uses the EZH2 (Enhancer of Zeste Homolog 2) active unit trimethylates histone H3 (tri-methylation of lysine 27 on histone H, H3K27me3) on the promoter region to silence the inflammatory gene into a heterochromatic state [16]. The inability of these components to work together might impair the whole molecular phase of the inflammatory genes.

### 2.2. Epigenetics of Epithelial–Mesenchymal Transition (EMT)

The succeeding proliferative stage involves a high level of lateral keratinocyte migration from the wound edge into the provisional matrix layer. The migration process requires a partially, reversible phenotype change called Epithelial–Mesenchymal Transition (EMT) [17].

During EMT, the differentiated keratinocytes lose their ability to interact via desmosome and adherens junction connections, becoming more mobile with a mesenchyme-like phenotype and higher contractile properties. The EMT process is tightly controlled by Snail (SNAI1), Slug (SLUG), and Twist transcription factors. Their activation relies upon chromatin remodeling [17].

To facilitate migration, the CDH1 gene that codes for E-cadherin is silenced via chromatin compaction mediated by an epigenetic mechanism [18]. The Snail protein associates with Sin3A (SIN3 transcription regulator family member A) and Class I HDAC to bind to the CDH1 promoter and remove acetyl group modifications from histones and proteins leading to the formation of a repressive complex. Simultaneously, the methyltransferase enzymes DNMT1 and DNMT3b (DNA methyltransferase 1 and DNA methyltransferase 3b) transfer methyl groups onto the CpG islands of the CDH1 gene [19].

On the other hand, the promoters that control SNAI1 and SLUG are regulated by the exact reverse modification process. These histone de-methylases like JMJD3/KDM6B (Lysine Demethylase 6B) can demethylate the histone at the level of the promoter region and remove these repressive marks like H3K27me3 from the promoter. On the contrary, these adjacent histones undergo fast acetylation mediated by histone acetyltransferases (HATs). In this state, the histone is opened for the recruitment of signaling mediators, including phosphorylated ERK or SMAD from the TGF-beta signaling pathway. After reaching the state of successful re-epithelialization and establishing the connection between cells, all this machinery gets turned back again [19].

### 2.3. Epigenetic Dysregulation Within the Multifactorial Microenvironment of Chronic Wounds

In chronic non-healing wounds, the persistent inflammatory microenvironment, local hypoxia, and high proteolytic activity interact with the cellular machinery, leading to dysregulated chromatin marks (often referred to as ‘epigenetic memory’).

While these chromatin alterations lock key repair genes, they act in synergy with extracellular stressors rather than as a standalone primary cause. Skin biopsy from chronic wounds indicates that the keratinocytes and dermal fibroblasts within the wounds become trapped in a pathologic state where they can no longer respond to stimuli to heal [20]. The enzymes involved in altering chromatin become dysregulated under chronic hyperglycemia or hypoxia, leading to the permanent locking of certain genes into open or closed structures.

An example of the loss of epigenetic regulation during wound healing is the chronic up-regulation of the NF-kB pathway. Within diabetic skin cells, high levels of glucose lead to the persistent activation of the methyltransferase Set7, which constantly marks the mono-methylation of lysine 4 on histone H3 (H3K4me1) in the promoter region of the RelA (p65) subunit of NF-kB. The constant epigenetic open structure allows for transcription of inflammatory mediators even when there is reduced glucose in the system [21,22].

Simultaneously, the repressive mechanism fails in stifling the inflammation-causing sites due to the repression of EZH2 expression, which occurs as a result of chronic oxidative stress in these ulcers. Thus, trimethylation of lysine 27 on histone H3 (H3H3K27me3) marks are not added to the promoters of inflammatory genes, resulting in the inability to condense chromatin [19].

On the other hand, the brakes on epigenetics are set up to prevent the functioning of all genes necessary for wound repair. In particular, the promoters of such growth factors as Vascular Endothelial Growth Factor (VEGF) and Transforming Growth Factor-beta 1 (TGF-beta1), as well as matrix metalloproteinases (MMPs), become excessively methylated by an excessively active DNMT1. Thus, no transcription factors can bind to them [23].

**Figure 1:**
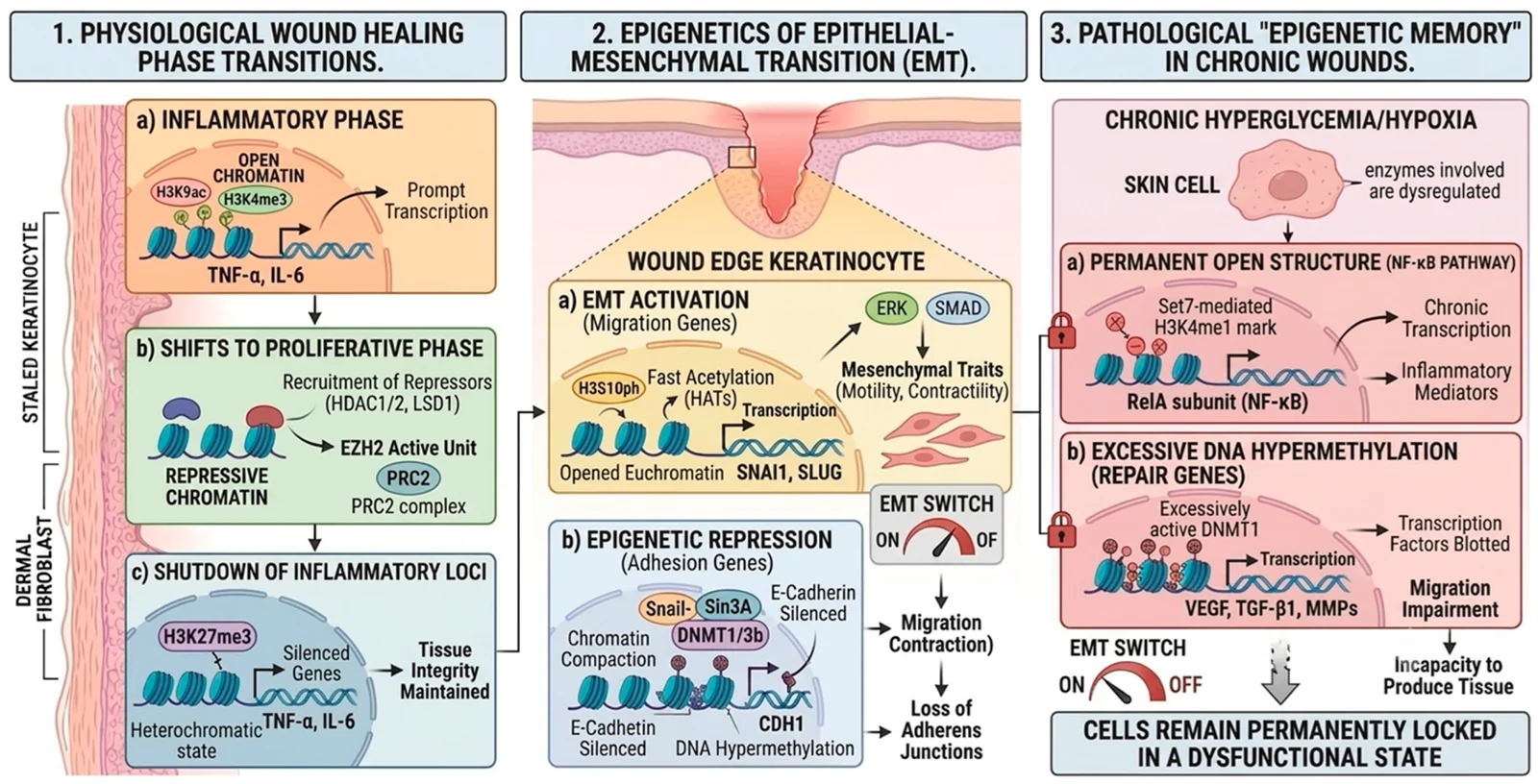
Epigenetic landscapes regulating physiological skin repair versus pathological chronic wounds. The schematic illustration delineates the dual chromatin dynamics in skin homeostasis and pathology. (**Left Panel**) Physiological Crosstalk: Epigenetic regulation of healing phases: Transition from the inflammatory to the proliferative phase requires active chromatin remodeling. Activating marks (H3K9ac, H3K14ac, and MLL1-deposited H3K4me3) drive prompt transcription of pro-inflammatory cytokines (TNF-α, IL-6). Resolution involves an epigenetic shutdown mediated by HDAC1/2, LSD1, and PRC2-dependent H3K27me3 deposition, promoting a heterochromatic state. (**Central Panel**) Epigenetics of EMT switch: Active keratinocyte migration depends on a reversible Epithelial–Mesenchymal Transition (EMT). Promoters of SNAI1 and SLUG are opened via H3S10ph and rapid acetylation driven by ERK/SMAD inputs, while the epithelial adhesion gene CDH1 (E-cadherin) is simultaneously silenced by the Snail-Sin3A-HDAC1 complex and DNMT1/3b-mediated hypermethylation. (**Right panel**) Pathological Block: Under chronic hyperglycemic or hypoxic conditions, cells develop a dysfunctional “epigenetic memory.” Chronic inflammation memory: Sustained glucose levels trigger overactivation of Set7, which locks the RelA promoter (NF-κB) in a permanently open H3K4me1 state, while oxidative stress suppresses EZH2, preventing chromatin condensation at inflammatory loci. Locked repair genes: Overactive DNMT1 drives aberrant DNA hypermethylation on essential regeneration genes (VEGF, TGF-β1, MMPs), blocking transcription factor binding and permanently trapping keratinocytes and fibroblasts in a non-healing, non-migratory state. Schematic layout initially drafted and refined using Gemini 3 Pro (Google).

Chromatin in dermal fibroblasts taken from these ulcers remains permanently condensed around promoters of genes necessary for collagen production and myofibroblast differentiation, leading to their migration impairment and incapacity to produce new tissue [24] (Figure 1).

## 3. Honey Bioactive Compounds as Direct and Indirect Epigenetic Modifiers

### 3.1. Manuka Honey vs. Non-Manuka Varieties

The vast majority of laboratory and clinical studies about therapeutic properties of honey involve Manuka honey, which is a monofloral type produced from Leptospermum scoparium [25]. This scientific bias centers around high methylglyoxal (MGO) content in Manuka and uses it as a main indicator of bioactivity and antibacterial properties.

Biochemically, MGO binds to proteins and generates advanced glycation end-products (AGEs), which in turn trigger intracellular signaling [26]. Still, the epigenetic impact of methylglyoxal on mammalian skin cells remains under heavy scrutiny.

Focusing strictly on Manuka restricts our knowledge about the whole spectrum of healing properties of honey. There are other types of honey, particularly those classified as dark honeys such as chestnut (*Castanea sativa*), forest, and specific monofloral honeys produced in Europe, which often demonstrate equally or better efficiency when they come to promoting wound healing [27,28].

Non-Manuka honey demonstrates unique composition with high amounts of polyphenols, flavonoids, and organic acids which function independently of MGO toxicity in order to eliminate the bacteria and activate healing processes [27]. Therefore, it can be concluded that true epigenetic modification capacity of honey is contained within its matrix rather than a single chemical compound.

Recent experimental evaluations confirm that non-Manuka dark honeys (such as chestnut and multifloral varieties) exhibit strong antioxidant and anti-senescence activities in skin-derived cell models through the direct preservation of chromatin-regulating factors [27]. Rather than relying on MGO-induced glycation stress, these natural matrices directly modulate cellular senescence markers and histone acetylation levels, proving that whole honey formulations exert direct epigenetic protective effects on somatic cells.

### 3.2. Polyphenols as DNMT and HDAC Inhibitors

While initial hypotheses relied on data from isolated phytochemicals, emerging experimental evidence directly demonstrates that whole honey matrices can elicit specific epigenetic responses in skin and somatic cells [29]. For instance, recent studies utilizing natural honey formulations have documented measurable changes in histone acetylation profiles, expression of chromatin-modifying enzymes, and protective epigenetic reprogramming against cellular senescence in stem cells and dermal models [30].

The polyphenolic fraction in honey is hypothesized to be the responsible for the chromatin-remodeling capabilities of honey [29] (as is seen in Table 1). The flavonoids chrysin, quercetin, and kaempferol, along with the phenolic acids caffeic acid, ferulic acid, and p-coumaric acid, function as direct small molecules responsible for the modulation of enzymatic proteins that regulate chromatin remodeling [29].

Specifically, it should be noted that the flavonoid quercetin and phenolic acid caffeic acid function as competitive inhibitors of DNMT1, DNMT3a, and DNMT3b isoforms of DNA methyltransferase enzymes [30]. The molecular mechanism of action of the above-mentioned polyphenols is the competitive binding with DNMT enzymes, thereby blocking the transfer of the methyl group from S-adenosylmethionine to the cytosine residue on DNA. In this manner, honey prevents the methylation of CpG islands in promoter regions of silent and regenerative genes.

Moreover, chrysin and apigenin can be considered natural inhibitors of HDACs, especially selective to class I and II HDAC enzymes [30,33].

As they bind the active site of HDAC enzymes, they hamper the process of deacetylation of the lysine residues on histone tails, especially in the case of histones H3 and H4. This is because this deacetylation hinders the maintenance of a negative charge on the histone proteins, which would keep them from being able to bond strongly with the negatively charged DNA molecule.

In this way, they maintain a state of chromatin, which enables the chromatin to remain open and relaxed in a process known as euchromatin. This state allows transcription factors access to the gene regions that determine the EMT as well as the proliferations of dermal fibroblasts. Thus, by inhibiting DNMTs and HDACs simultaneously, honey polyphenols serve as active chromatin remodeling agents for the regeneration of skin cells [38].

### 3.3. Honey-Derived MicroRNAs (XenomiRs): Opportunities and Debates

Apart from common low molecular weight polyphenolic compounds, the epigenetic capability of honey is strongly dependent on its stable non-coding RNA component. This stable fraction of RNAs mainly comprises plant-derived miRNAs, also known as xenomiRs [39]. This family of RNA consists of small RNAs (mainly 19–24 nucleotides in length), which were directly extracted from the nectar and pollen of honey plants, but some components can be acquired from honeybees (*Apis mellifera*) when converting floral nectar into honey [40].

One of the most important properties of the miRNA fraction in honey is their surprising structural stability while existing in the natural matrix of honey. Raw honey is considered a chemically aggressive medium characterized by acidic pH level (from 3.2 up to 4.5), high osmotic pressure and the presence of many active endogenous enzymes. However, despite all these factors, not only does the miRNA fraction retain its biological functions, but it also remains undegraded throughout the period of prolonged honey storage [41].

According to biochemical studies, this surprising stability can be explained by their natural packaging, as the xenomiR fraction is either contained in the lipid membrane of exosomes or associated with the protein argonaute complex to prevent degradation by RNases [39].

The application of honey on the compromised wound bed causes these lipid vesicles to fuse with the membranes of the mammalian cells, which allow the uptake of the xenomiRs by the keratinocytes and dermal fibroblasts through endocytosis. When the plant xenomiRs have been internalized, they enter the mammalian cytoplasm, bypassing the nuclear DNA transcription process. Instead, the post-transcription process becomes the target because the complementary mRNAs get bound by these foreign RNA molecules [39].

Through the cross-kingdom complementary interaction of the RNA molecules, the mRNAs of mammalian cells become targeted for mRNA degradation or blockage of translation into protein. Consequently, post-transcription gene silencing is induced. Using next-generation sequencing techniques, specific microRNAs have been identified in honey, including miR-156 and miR-162 [41].

These xenomiRs can specifically target the mammalian signaling nodes involved in the inflammatory cascade through the binding to the 3′ untranslated region (3′-UTR) of mRNAs of pro-inflammatory cytokines (TNF-α) [41]. As a result, they promote the transition of chronic inflammation to the proliferative phase.

Although next-generation sequencing has identified specific plant microRNAs in honey (such as miR-156 and miR-162) and in silico predictions suggest potential sequence complementarity with mammalian inflammatory transcripts (e.g., TNF-α), these interactions remain purely hypothetical. Substantial experimental validation, demonstrating that exogenous xenomiRs can survive the protease- and RNase-rich environment of chronic wound exudates to achieve meaningful post-transcriptional gene silencing in vivo, is strictly required before assigning a definitive therapeutic role to this fraction.

## 4. The Nuclear Bridge: Crosstalk Between Cellular Signaling and Chromatin Remodeling

### 4.1. H_2_O_2_ Influx via Aquaporins and Epigenetic Readout

The initiation process of wound healing by honey involves a brief period of mild and localized oxidative stress [42]. This event represents a tightly regulated form of cellular signaling called oxidative eustress. Honey, which contains a low amount of hydrogen peroxide (H_2_O_2_) produced enzymatically by glucose oxidase from bees’ enzymes, produces constant flow of hydrogen peroxide in wounds after dilution in the wound exudates [43].

These peroxides, together with natural peroxides and polyphenols present in the medium, act as a very important second messenger signal.

Unlike diffusion of the molecule slowly through the lipid bilayer, the peroxide signaling system is transported quickly across mammalian cell membranes by the channel-mediated mechanism in target cells such as basal keratinocytes and dermal fibroblasts, mainly through Aquaporin-3 (AQP3), peroxiporin channels expressed at high levels in epidermal cells plasma membranes [9].

Once H_2_O_2_ enters the intracellular compartment, it changes the local redox environment, oxidizing low-pKa residues, specifically the Cys124 catalytic site in protein tyrosine phosphatases, including PTP1B and PTEN, in a highly selective and reversible manner [43].

The temporary inhibition of such enzymes prevents the dephosphorylation of the upstream receptor tyrosine kinases. Therefore, this leads to an extended activation of the downstream survival and migratory signaling pathways such as PI3K/Akt and MAPK/ERK signaling pathways [10].

Moreover, it should be noted that molecular effects of such an aquaporin-mediated influx go far beyond simple intracellular signaling cascades and involve direct nuclear epigenetic modifications. The transient increases in the levels of intracellular H_2_O_2_ pass through the nuclear membrane and alter nuclear metabolism by altering the ratio between oxidized and reduced nicotinamide adenine dinucleotide (NAD+/NADH) [44]. This NAD+/NADH ratio becomes a direct structural rheostat for NAD+-dependent Class III Sirtuin HDACs such as SIRT1 and SIRT6 [45].

Thus, due to the temporary increase in the levels of NAD+ in the nucleus, the activity of the Sirtuins is upregulated and their binding affinity and the rate of acetylation/deacetylation of certain residues become different. For instance, the enzymatic activity of SIRT1 is directed to the removal of the acetylation marks of lysine residues on histone H3 (H3K9 and H3K56) [46].

The remodeled chromatin structure changes the accessibility to a certain structure of promoters. Such opening results in quick recruitment of RNA polymerase II at certain sites required for the antioxidant response and cell survival [47].

**Figure 2:**
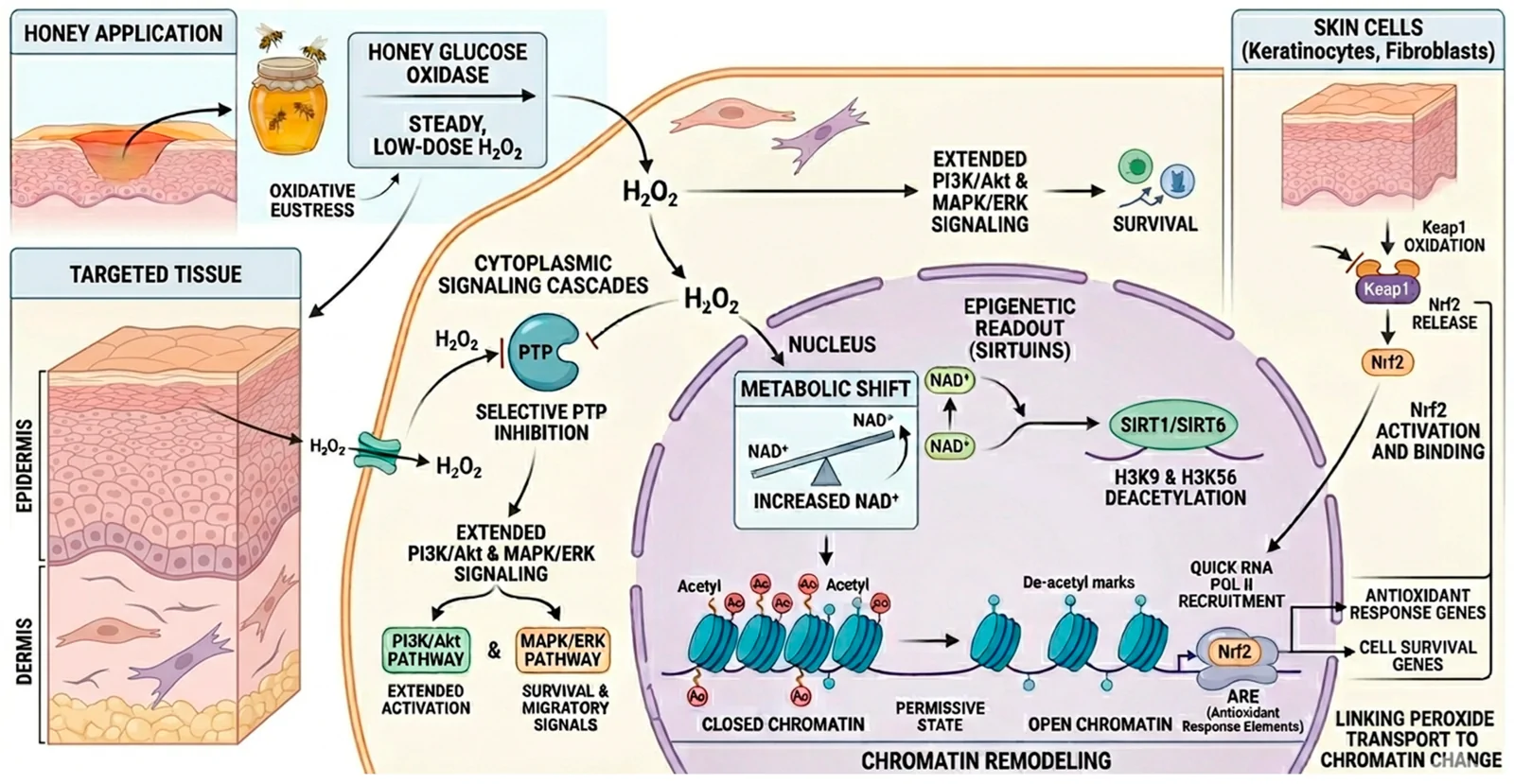
Honey-Derived H_2_O_2_ and Nucleocytoplasmic Signaling. Schematic overview of the unified pathway by which honey matrix glucose oxidase generates low-dose hydrogen peroxide (H_2_O_2_). The peroxide signal rapidly modulates protein tyrosine phosphatase (PTP) activity, while simultaneously shifting the nuclear metabolic ratio (Metabolic Shift) to activate SIRT1/SIRT6. In parallel, H_2_O_2_ oxidizes Keap1, triggering Nrf2 nuclear entry. Convergent signaling facilitates chromatin remodeling and the sequential upregulation of essential cell survival and antioxidant genes required for dermal remodeling. Schematic layout initially drafted and refined using Gemini 3 Pro (Google).

At the same time, such a process of oxidation of the histone molecules works in tandem with oxidation of non-histone substrate targets. One example would be the oxidation of Keap1 (Kelch-like ECH-associated protein 1), followed by nuclear translocation of Nrf2 (Nuclear Factor Erythroid 2-related factor 2) [48]. The latter binds with Antioxidant Response Elements (ARE), which has been previously unveiled due to chromatin remodeling. Such a combination establishes a direct mechanical link between the channels mediating peroxide transport and chromatin remodeling [49] (Figure 2).

### 4.2. Calcium (Ca^2+^) Transients and Histone Modifications

In conjunction with the redox signaling axis, topical application of honey induces a fast and transient increase in the concentration of intracellular calcium (Ca^2+^) ions [9]. Unlike the redox signaling upsurge, the intracellular calcium wave is generated by an elaborate biphasic process that includes immediate mobilization of the existing intracellular Ca^2+^ pools in the lumen of the endoplasmic reticulum (ER), induced through activation of IP3 receptors, and sustained entry of external Ca^2+^ through plasma membrane Store-Operated Calcium Entry (SOCE) [50].

The latter component of the intracellular wave of calcium ions appears to be directly associated and tightly coupled with the second phase of redox signaling in that the onset of the calcium influx occurs downstream of an oxidative AQP3-induced signaling wave and is dependent on the opening of calcium channels due to peroxide fluxes as its initiating factor [10].

Rapid transients in the concentration of intracellular Ca^2+^ ions are sensed by keratinocytes and fibroblasts through calmodulin, activating Calcium/calmodulin-dependent protein kinase II (CaMKII) and MAPK/ERK cascades for cell proliferation and migration [51].

The connection between these short-term Ca^2+^ signals in the cytosol and lasting changes in the gene expression is made by an intricate process involving two actions at the nuclear level. The dual action includes both the direct phosphorylation of histones as well as the actual removal of large transcriptional inhibitors from the nucleus [52]. ERK and CaMKII quickly pass through the nuclear membrane, where they target by phosphorylation 10th amino acid (serine) on the histone H3 protein (histone H3S10ph)—a hallmark of immediate early gene expression [53].

This specific type of post-translational modification adds large, negative phosphate groups to histone tails. From a structural perspective, the negative charge of phosphate groups pushes away the similarly charged DNA backbone, thereby relaxing the tightly wound chromatin. This opens the structure and makes it possible for histone acetyltransferases (HATs), such as the p300/CBP complex [54].

This cooperative histone acetylation, enabled by its previous phosphorylation, immediately activates the promoters of immediate early genes, including key transcription factors involved in EMT—such as SNAI1 (Snail), SLUG, and some important matrix metalloproteinases (such as MMP-2 and MMP-9) [55,56].

At the same time, the honey-derived calcium influx influences the phosphorylation status of Class IIa histone deacetylases (e.g., HDAC4 and HDAC5) [56]. In quiescent or chronically wounded skin cells, these histone deacetylases are localized in the nucleus and associated with transcriptional co-repressors such as NCoR (Nuclear Receptor Corepressor 1) and SMRT (Silencing Mediator for Retinoid and Thyroid Receptors) that maintain the condensed state and gene silencing of wound-healing genes [57].

The activation of nuclear calcium/calmodulin-dependent protein kinase (CaMK) enzymes via calcium results in the phosphorylation of serine residues of the HDAC proteins. Such a phosphorylation process generates distinct sites for association with chaperone 14-3-3 proteins, which initiates their subsequent nuclear export into the cytosolic compartment. The nuclear export is performed by an active process called CRM1-mediated export [58].

In this way, the transcriptional repression of wound-healing genes by such repressive complexes becomes impossible.

## 5. Future Perspectives and Clinical Implications

### 5.1. Nutriepigenetic Perspectives and Hypothesized Chromatin Modulation

The clinical management of chronic, non-healing ulcers—ranging from diabetic foot complications to venous stasis wounds—remains a major therapeutic challenge. Current standard treatments are largely reactive, focusing on physical debridement, maintaining a moist environment, and managing local infections [59].

While these steps are necessary, they completely fail to address the underlying molecular pathology: a severely dysregulated and locked gene expression profile within the wound cells. The biochemical data compiled in this review pointing toward a nutriepigenetic approach suggests a completely different clinical strategy. Instead of applying a single synthetic drug targeting an isolated receptor pathway, the topical application of honey could provide a multi-component natural matrix capable of hitting several epigenetic checkpoints simultaneously.

It is crucial to maintain a clear delineation between experimentally validated in vitro biochemical events—such as isolated polyphenol inhibition of HDACs or H_2_O_2_-induced calcium fluxes—and speculative in vivo outcomes in intact chronic wound beds. While these cellular pathways provide plausible mechanistical bridges, their actual capacity to induce physiological chromatin remodeling in human non-healing ulcers remains to be proven.

Direct experimental evidence confirms that honey exposure modulates key epigenetic targets—such as altering DNMT levels, modulating HDAC activity, and regulating H3K9/H3K14 acetylation in cell culture and skin repair models [31,36,38]. However, a clear distinction must be maintained between these validated in vitro/ex vivo biochemical events and full in vivo chromatin remodeling within complex human chronic wound beds. While these cellular data prove that honey bioactives directly engage nuclear chromatin machinery, establishing the exact kinetics, bioavailability, and depth of tissue reprogramming in human diabetic or venous ulcers remains an ongoing clinical challenge.

The immediate goal from a clinical perspective is to use this complex mixture to actively “reset” the locked chromatin state that characterizes diabetic keratinocytes and senescent fibroblasts. In these compromised cells, the chromatin is physically closed around essential healing loci. Honey components could change this architecture.

By stimulating the nuclear export of Class IIa HDACs and blocking overactive DNMT enzymes, topical honey application can lift the epigenetic brake that stalls tissue repair.

This multi-target action allows the cell to re-open closed chromatin domains, turning back on the expression of silenced genes responsible for cell migration, angiogenesis, and extracellular matrix deposition.

Instead of deterministic molecular reprogramming, topical honey application is best conceptualized as a putative bio-active matrix. The proposed model suggests that multi-component interactions might help attenuate the repressive chromatin environment in stalled cells. However, whether these localized biochemical signals translate into verified in vivo tissue reprogramming remains a hypothesis requiring experimental validation.

In summary, while the direct capacity of honey components to alter HDAC/DNMT activity and modify histone acetylation marks is now firmly validated in experimental cell models [31,36,38], translating these findings to intact human wounds requires moving from established in vitro proof-of-concept to targeted in vivo chromatin mapping.

### 5.2. Limitations and Challenges in Standardizing Epigenetic Therapies

Introducing such innovative honey-mediated nutriepigenetic intervention into common clinical protocols poses various challenges. First, it should be considered that the chemical composition of the honey matrix per se is rather inconsistent. Unlike a stable pharmaceutical compound, it changes depending on the geographic origin, composition of the soil, plant species used, seasonal characteristics, and even storage conditions after harvesting.

Therefore, the amounts of specific epigenetic modulators that are being delivered will also vary. Such a biochemical inconsistency does not allow formulating standardized dosage protocols needed for conducting rigorous randomized clinical trials.

A further major challenge concerns the highly controversial status of cross-kingdom gene regulation by dietary-, plant-, or bee-derived microRNAs (xenomiRs) [41]. While the presence of small RNA fragments in honey has been documented, biological cross-kingdom delivery remains widely debated in the literature, with significant conflicting reports regarding functional uptake efficiency and physiological relevance in host tissues.

In the context of non-healing ulcers, there is currently a total lack of definitive proof that honey-derived xenomiRs can survive intact within extracellular vesicles or protein complexes when exposed to the high concentration of endogenous proteases, nucleases, and active RNases present in chronic wound exudates.

Furthermore, cellular uptake kinetics, intracellular stability, and direct gene-silencing targeting by these xenomiRs in target keratinocytes or dermal fibroblasts in vivo represent a speculative secondary hypothesis rather than an established driver of wound healing. These mechanistic connections remain unproven knowledge gaps that demand rigorous experimental verification before xenomiRs can be considered viable therapeutic modalities.

Regarding the latter issue, it is essential to overcome the limitations of current approaches that are mostly limited to in vitro assays, such as the scratch test in terms of mechanistic and time-resolved understanding of the impact of a topical application of honey on an organism’s physiology. Thus, it is essential to use ChIP-Seq combined with single-cell RNA sequencing in appropriate models to evaluate precisely the changes in histone modifications and chromatin accessibility caused by honey.

Furthermore, the clinical efficacy of any nutriepigenetic strategy is intrinsically constrained by inter-individual heterogeneity. Factors such as patient age, underlying metabolic dysfunction (e.g., severe hyperglycemia), local vascularization, and the composition of the wound microbiome create highly variable epigenetic landscapes across patients.

Consequently, honey-mediated nutriepigenetic modulation cannot be viewed as a ‘one-size-fits-all’ solution. Instead, future translational research must evaluate how specific patient endotypes respond to standardized medical-grade honey formulations, paving the way for biomarker-guided, personalized wound management.

Moreover, a crucial consideration in modulating epigenetic mechanisms, such as HDAC inhibition, SIRT activation, and chromatin remodeling, is the potential risk of oncogenic transformation, as these pathways are frequently dysregulated in tumorigenesis.

However, a clear distinction must be made between systemic, high-dose pharmacological epigenetic drugs and the localized, low-dose, physiological action of topical medical-grade honey. Honey-mediated chromatin relaxation operates as a transient, self-limiting homeostatic shift that allows quiescent, impaired keratinocytes to re-enter the physiological cell cycle and migration programs, without driving genomic instability.

**Figure 3:**
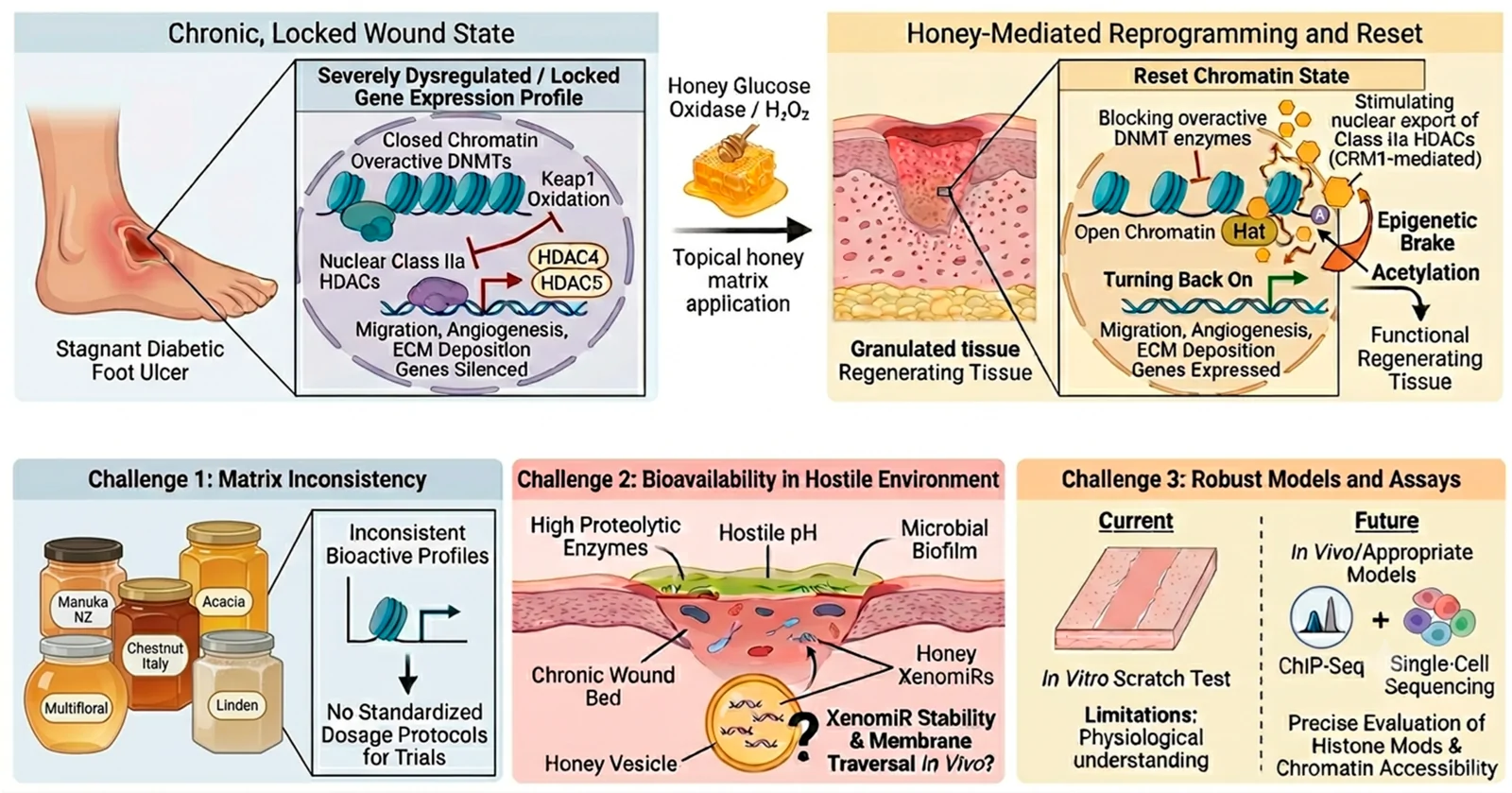
Future translational perspectives and biochemical challenges of honey nutriepigenetics. (**Top Panel**) Epigenetic Reset/Chromatin Modulation: Chronic, stagnant wounds (e.g., diabetic foot ulcers) are characterized by a locked chromatin state where overactive DNMTs and nuclear Class IIa HDACs (HDAC4/5) silence key angiogenic and migratory loci. Topical application of the multi-component honey matrix resets this architecture by blocking DNMTs and inducing CRM1-mediated nuclear export of HDACs, opening chromatin domains to transform stagnant tissue into functional regenerating tissue. (**Bottom Panel**) Clinical Obstacles: Translation into standard clinical protocols faces three core challenges: (Challenge 1) Matrix inconsistency across floral and geographical origins, preventing standardized trial dosages; (Challenge 2) Unknown bioavailability and stability of fragile honey xenomiRs against the hostile, proteolytic, and biofilm-rich chronic wound bed; and (Challenge 3) The current reliance on basic in vitro scratch assays, highlighting the critical future need for in vivo single-cell RNA-seq and ChIP-Seq profiling to precisely map chromatin accessibility. Schematic layout initially drafted and refined using Gemini 3 Pro (Google).

Additionally, extensive preclinical literature indicates that honey bioactives exert selective pro-apoptotic and anti-proliferative effects specifically in transformed cells while preserving normal tissue architecture. Nevertheless, long-term safety studies in chronic wound models remain essential to monitor tissue homeostasis (Figure 3).

## 6. Conclusions

The evolution of chronic wounds from a reactive approach aimed at dressing the wound to an active approach where nucleocytoplasmic events are involved represents a major step in the management of such conditions.

The evolving understanding of chronic wound management highlights the complex interplay between the local microenvironment and cellular signaling pathways. The stalled cellular phenotype observed in non-healing ulcers is a multifactorial state wherein epigenetic dysregulation represents one interactive regulatory layer.

Medical-grade honey offers a rich source of bioactives that may serve as a putative nutriepigenetic matrix, interacting with cellular pathways via transient redox signals and ionic fluxes in vitro. However, concepts proposing honey as a targeted ‘epigenetic editor’ or a direct tool for tissue ‘reprogramming’ overstate current scientific evidence.

Future research utilizing single-cell transcriptomics and ChIP-Seq in robust in vivo models will be essential to establish whether these biochemical interactions can deliver clinically meaningful chromatin modulation in human wound healing.

## Figures and Tables

**Table 1:** Direct and indirect epigenetic modalities of honey bioactive components in wound tissue repair. The table summarizes the targeted chromatin-modifying enzymes, core molecular mechanisms, and downstream cellular phenotypes driven by major low-molecular-weight phytochemicals and non-coding RNA fractions found across distinct honey varieties.

Compound/Honey Type	Epigenetic Target	Molecular Mechanism	Cellular Outcome in Wound Healing	References
Methylglyoxal (MGO) (*Manuka Honey*)	DNMT1/Histone core	Downregulation of DNMT1 expression; potential direct glycation of histone tails.	Modulation of keratinocyte proliferation and suppression of hyper-inflammatory genes.	[31,32]
Quercetin (*Monofloral & Dark honeys*)	DNMT1, DNMT3a, DNMT3b	Competitive inhibition of the DNMT catalytic site; blocks DNA hypermethylation.	Re-activation of silenced migration genes; stimulation of keratinocyte scratch closure.	[30]
Chrysin (*Propolis & Honey matrix*)	Class I/II HDACs (HDAC1, HDAC3, HDAC8)	Direct enzymatic inhibition of HDACs, maintaining histone H3/H4 hyperacetylation.	Chromatin relaxation at *SNAI1* and *SLUG* promoters; induction of EMT traits.	[33,34]
Caffeic Acid (CAPE) (*Chestnut & Dark honeys*)	DNMT1/HDAC1	Non-competitive inhibition of DNMTs; epigenetic silencing of NF-kB targets.	Reduction in pro-inflammatory cytokines; shift toward fibroblast activation.	[35]
Apigenin (*Wildflower & Lime honeys*)	HDAC2/Histone H3	Inhibition of deacetylase activity; promotes localized H3K9ac and H3K14ac accumulation.	Upregulation of matrix metalloproteinases (MMPs); facilitates dermal remodeling.	[36]
Honey XenomiRs (*Plant-derived microRNAs*)	Target mRNAs (Post-transcriptional)	Sequence-specific binding to 3′-UTR of mammalian mRNAs, bypassing transcription.	Downregulation of inflammatory checkpoints; acceleration of re-epithelialization.	[37]

## Data Availability

No new data were created or analyzed in this study. Data sharing is not applicable to this article.

## Abbreviations

The following abbreviations are used in this manuscript:

AQP3	Aquaporin-3
ARE	Antioxidant response element
BMAL1	Brain and muscle Arnt-like protein 1
CaMKII	Calcium/calmodulin-dependent protein kinase II
CAPE	Caffeic acid phenethyl ester
CBP	CREB-binding protein
ChIP-Seq	Chromatin immunoprecipitation sequencing
CLOCK	Circadian locomotor output cycles kaput
CRM1	Chromosomal maintenance 1 (Exportin-1)
CRY1	Cryptochrome 1
Cys	Cysteine
DFU	Diabetic foot ulcer
DNA	Deoxyribonucleic acid
DNMT	DNA methyltransferase
ECM	Extracellular matrix
EMT	Epithelial–mesenchymal transition
ERK	Extracellular signal-regulated kinase
FTIR	Fourier-transform infrared spectroscopy
HAT	Histone acetyltransferase
HDAC	Histone deacetylase
HPP	High-pressure processing
H_2_O_2_	Hydrogen peroxide
IP3	Inositol trisphosphate
KEAP1	Kelch-like ECH-associated protein 1
LNP	Lipid nanoparticle
MAPK	Mitogen-activated protein kinase
MGO	Methylglyoxal
miRNA	MicroRNA
MMP	Matrix metalloproteinase
NAD+	Nicotinamide adenine dinucleotide (oxidized)
NADH	Nicotinamide adenine dinucleotide (reduced)
NCoR	Nuclear receptor co-repressor
NMR	Nuclear magnetic resonance
NRF2	Nuclear factor erythroid 2-related factor 2
PER2	Period circadian protein 2
PI3K	Phosphoinositide 3-kinase
PTEN	Phosphatase and tensin homolog
PTP	Protein tyrosine phosphatase
RNA	Ribonucleic acid
SIRT	Sirtuin (NAD+-dependent deacetylase)
SLUG	Snail family transcriptional repressor 2
SMRT	Silencing mediator for retinoid and thyroid hormone receptors
SNAI1	Snail family transcriptional repressor 1
SOCE	Store-operated calcium entry
SOCs	Store-operated calcium channels
xenomiR	Plant-derived xenomiR (microRNA)
UTR	Untranslated region
